# Spontaneous Rupture of Malarial Spleen: Report of Two Cases

**DOI:** 10.4084/MJHID.2010.036

**Published:** 2010-12-13

**Authors:** M Ezzedien Rabie, Ahmad Al Hashemey, Ismail El Hakeem, Mohammad Ali Al Hakamy, Mahmoud Obaid, Mohammad Al Skaini, G Shabbir, Saeed Al Sareii, Mir Najeeb Hussain

**Affiliations:** 1 Department of surgery and; 2 Medicine-The Military Hospital, Southern Region-Khamis Mushait-Saudi Arabia

## Abstract

Malaria is endemic in many tropical and subtropical regions of the world, including Saudi Arabia. The infection has serious consequences in those residing in non endemic regions on travelling to endemic areas, due to lack of immunity to the parasite. In this report, we describe the clinical course of two patients who travelled to a malaria endemic area. Both contracted the infection and presented with splenic rupture. They received splenectomy in addition to the appropriate antimalarial medications, with successful outcome.

## Introduction:

Malaria is a life-threatening endemic disease in over 100 tropical and subtropical countries, visited by more than 125 million travelers yearly. The human variant is caused by the protozoan parasite Plasmodium of which four spices are known: falciparum, malariae, ovale and vivax, transmitted by female Anopheles mosquitoes.^1^

The risk of contracting malaria, mainly due to Plasmodium falciparum, is present all year round in most of the south western region of Saudi Arabia, including Jazan area and the lowlands of Aseer region (Tehama), while excluding its mountainous parts. In these endemic regions, chloroquine-resistant Plasmodium falciparum has been reported and preventive measures for incoming travelers entail type IV prophylaxis, which includes mosquito bite prevention in addition to mefloquine, doxycycline or atovaquone/proguanil.^1^

In this report, we highlight the possibility of splenic rupture in the course of a malarial episode, particularly in individuals residing in non epidemic areas who travel to a malaria endemic area.

## Case 1:

26-year-old male, presented with fever, rigors, profuse night sweating, and mild epigastric pain, for three days. His past history was positive for a recent travel to Jazan area, and there was no history of recent trauma. On examination, he looked ill, his blood pressure was 108/68 mm Hg, pulse 130/min and temperature 38.9 °C. Chest examination showed good bilateral air entry, and abdominal examination revealed mild epigastric tenderness.

His white cell count was 4000 /mm^3^, Hb was 15.3 gm/dl and platelet count was 32000/mm^3^. Apart from low potassium level (2.9 mEq/dl, reference range 3.5–5.5 mEq/dl), his metabolic and liver panels were normal. A peripheral blood smear confirmed infection with plasmodium falciparum and antimalarial treatment was started, which consisted of 600 mg of intravenous quinine dihydrochloride every 8 hours, in addition to doxycycline 100 mg once a day orally for seven days.

**Figure 1.**
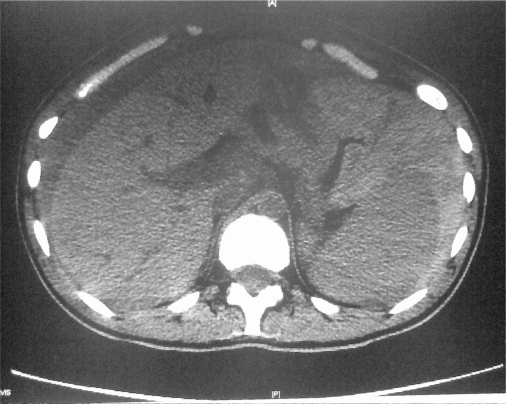
CT scan of the abdomen showing free fluid surrounding the liver.

Two days after admission, abdominal pain increased and became generalized, his blood pressure dropped to 88/44 mm Hg, his pulse was 137 /min and his temperature was 38.8 °C. Abdominal examination showed generalized tenderness, guarding with rebound tenderness. His haemoglobin dropped to 5.8 gm/dl and ultrasound/computerized axial tomographic examination showed significant intra abdominal fluid but failed to localize its source (Figure 1).

**Figure 2.**
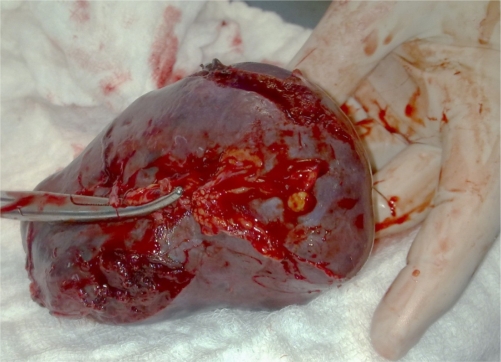
Splenic bursting tears near the upper and lower poles.

The patient was resuscitated with intravenous fluids, inotropes, blood and platelet transfusions. Laparotomy was conducted through a midline incision and intraperitoneal blood and clots were evacuated. The bleeding source was found to be the spleen with bursting tears at the upper and lower poles (Figure 2). Splenectomy was performed and a drain was inserted in the splenic bed.

Post operatively, the patient received antipneumococcal vaccine and the recovery period was uneventful, apart from copious discharge of serous fluid, which gradually decreased and the drain was timely removed. The patient was discharged after full recovery for follow up.

On careful enquiry, it was clarified that the patient was non compliant with the antimalarial prophylaxis provided by the medical authorities.

## Case 2:

A 28-year-old male, presented to the emergency room with left lumbar pain, radiating to the infra umbilical region, associated with high fever and once vomiting. His illness started four days previously and there was no other associated symptoms. His history included a recent stay in Jazan area for three months.

On examination, he was not pale, cyanosed nor jaundiced. His pulse was 90/minute, blood pressure 83/52 mm Hg, temperature 38.5 °C and oxygen saturation 92% on room air. His chest and heart examination were unremarkable and there was mild tenderness in the left lumbar and hypochondrial regions.

**Figure 3.**
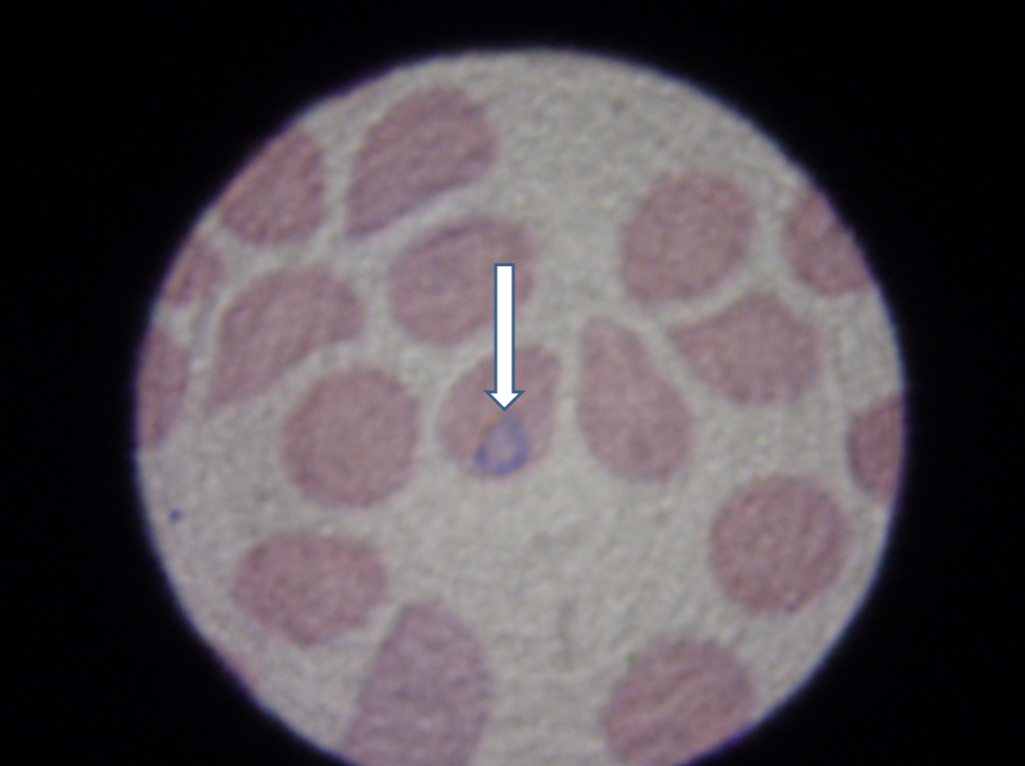
Microphotograph of a blood film for malaria. The arrow points to the intracellular parasite (H&E, oil immersion, ×100, magnified view)

His white cell count was 3000/mm^3^, Hb 12.4 gm/dl, platelet count 100,000 mm^3^, bilirubin 74 μmol/l (reference range 5.1–17.0 μmol/l), gamma glutamyl transferase 172 u/l (reference range 0 to 51 iu/l), alkaline phosphatase 137 iu/l (reference range 30 to 120 iu/l), alanine aminotransferase 199 u/l (reference range 9 to 60 iu/l). His renal values and electrolytes as well as his international normalized ratio were within normal. Due to the suggestive history and presentation, a blood film for malaria was requested and came positive (Figure 3).

**Figure 4.**
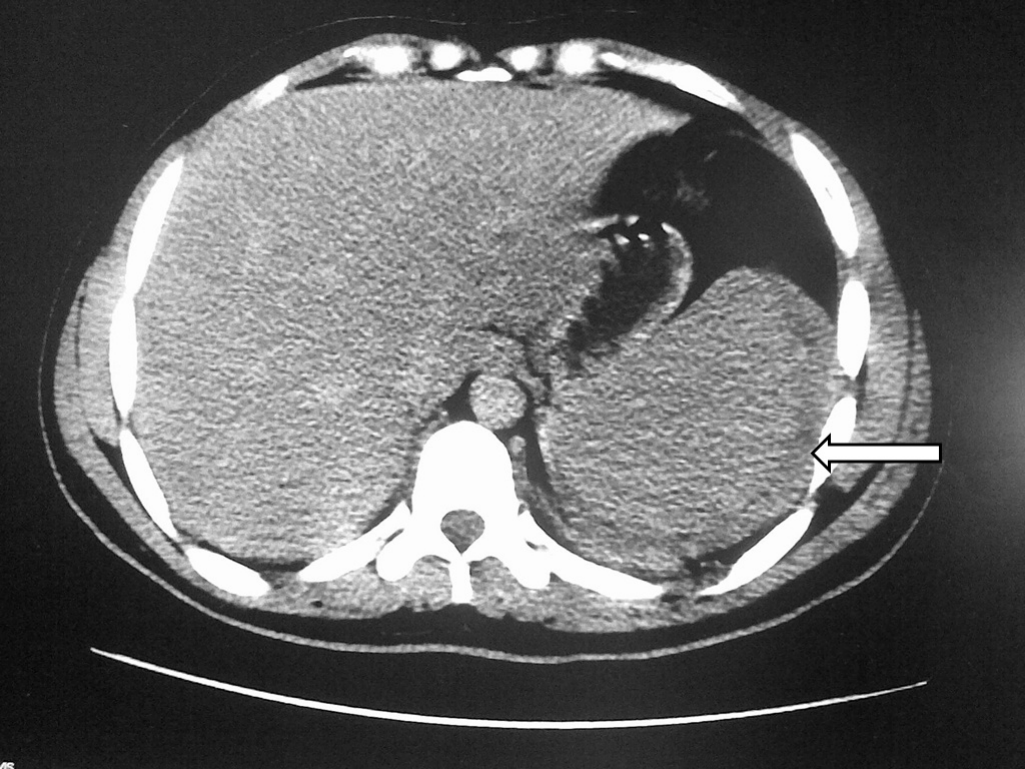
Free fluid surrounding the enlarged spleen (arrow).

Ultrasound/computerized axial tomography of the abdomen showed enlarged spleen with free fluid in the peritoneal cavity (Figure 4).

**Figure 5.**
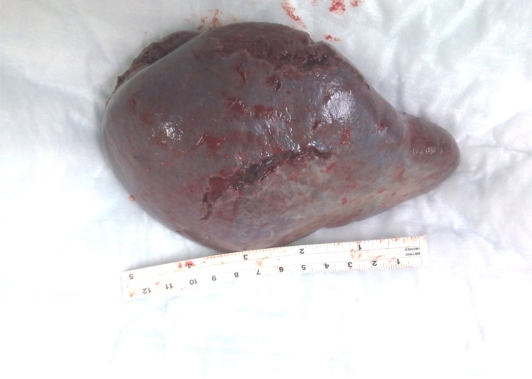
Capsular tear on the diaphragmatic surface of the spleen.

On emergency laparotomy, gross amount of blood was found in the peritoneal cavity with a 4 cm tear on the diaphragmatic surface of the spleen (Figure 5). Splenectomy was done and suction drain inserted. On recovery from anaesthesia and attempted extubation, the patient passed into respiratory distress and was reintubated. Chest X ray showed bilateral pulmonary infiltrates and he was shifted to the intensive care unit where anti pneumococcal vaccine was given together with the antimalarials quinine dihydrochloride 600 mg intravenously every 8 hours and doxycycline 100 mg PO OD, for seven days.

The differential diagnosis of the chest condition included acute lung injury and non cardiogenic pulmonary oedema. The patient responded to supportive treatment and his respiratory parameters and chest X ray gradually improved and he was extubated on the 5^th^ post operative day. He was eventually discharged in a good condition for follow up.

On enquiry, the patient admitted that he was non compliant with the antimalraial prophylaxis provided by the medical authorities.

## Discussion:

Plasmodium falciparum is responsible for the severest form of malaria, the clinical features of which may vary from fever, chills, headache, and muscular aches to acute renal failure, generalized convulsions, circulatory collapse, coma and death. Individuals residing in endemic areas acquire immunity against the parasite during intra uterine life.^2^ Their Immunity may wane or disappear if they leave their abode for more than six months. A mortality rate of 1% has been reported in inhabitants of endemic areas due to the parasite, with a much higher rate among incoming travelers who lack the immunity.^1^

Plasmodium falciparum is endemic in certain regions in Saudi Arabia, including Jazan area,^1^,^3^ where a rate of chloroquine resistant Plasmodium falciparum of 89.5% has been reported, the highest in the kingdom.^4^

Splenic reactions to malarial infection include splenomegaly, haematoma formation,^5^ splenic infarction^6^ and, rarely, abscess formation.^7^,^8^

The exact mechanism of splenic rupture is not fully understood. Three different mechanisms are thought to be involved. Firstly, cellular hyperplasia and venous-sinusoidal engorgement leading to increased tension in the organ. Secondly, vascular occlusion by reticulo-endothelial hyperplasia leading to thrombosis and infarction. Thirdly, episodic increase in the intra abdominal pressure by activities such as sneezing, coughing and defecation, compresses the tense organ. Acting together, these factors bring about parenchymal and subcapsular haemorrhages which strip the capsule, leading to further subcapsular haemorrhage. Eventually, the capsule tears leading to free intraperitoneal haemorrhage. (Hershey and lubitz).^9^ In chronic malaria, the splenic enlargement is gradual and tension within the capsule is less pronounced. For that reason, splenic rupture in such cases is less frequent.^9^

The clinical features of splenic rupture fall into two main categories: circulatory compromise due to loss of volume and abdominal signs due the presence of free intraperitoneal bleeding.^9^ Although the majority of cases with splenic rupture have peritoneal signs, some cases may have a subtle presentation.^10^ This fact should be remembered when dealing with a malarial patient who develops hypotension.

Travelling to malaria endemic areas mandates antimalarial prophylaxis. For Saudi Arabia, this entails type 4 prophylaxis.^1^ Despite the antimalarial prophylactic medications our patients were given, they were non compliant in taking the drugs. In this respect, it is worth mentioning that despite its importance, prophylactic treatment does not totally eliminate the possibility of contracting the disease^1^.

Non operative management for splenic rupture in malaria has received attention.^11^,^12^ However, this demands repeated transfusions, ready access to serial radiologic assessment, diligent follow up and preparedness to operate, no mater when, once deterioration occurs. All this may pose certain strains on the medical caregivers. The acute phase is certainly made easier by performing splenectomy and solving a life threatening component of the patient’s multi medical problems, while concentrating on the others.

In our previous work, we questioned the advisability of splenectomy for patients residing in malaria endemic areas, who contract splenic hydatid disease.^13^ The rationale behind that is the importance of the immune functions of the spleen in the fight against malaria. Compared to normal individuals, asplenic patients living in malaria-endemic areas are more commonly parasitaemic, and have delayed clearance of the parasite after treatment.^14^,^15^ Additionally, the disease may be severe or even fatal in these individuals.^16^ For this, it has been suggested that asplenic patients residing in malaria-endemic areas should consider lifelong prophylaxis against the parasite.^17^ Fortunately, this does not apply to our patients as both reside in non endemic areas.

In a recent review, the treatment offered, whether splenecotmy or conservative management, had no effect on prognosis^12^. Current knowledge about spontaneous rupture of the malarial spleen has been gained largely through case reports. A clear answer to which treatment is better, could be obtained only through randomized controlled trials, which are impossible to be carried out for obvious reasons. In this review, Imbert et al retrieved only 55 cases of malarial splenic rupture, published in the last 50 years in five languages The exact number should be much higher, as an old review in 1948 by Hershey and Lubitz reported 64 cases from 1917 onwards.^9^ Before 1917, seventy two cases were also reported by Leighton.^18^

Information about risk factors for splenic rupture in such cases is missing and lack of previous immunity to the parasite appears to be the only recognized one.^19^

## Conclusion:

Rupture of the malarial spleen is an underreported serious illness, which may affect travelers to endemic areas. Precautions against mosquito bites and appropriate antimalarial prophylaxis should be carefully adhered to. Particularly for those residing in non endemic areas, splenectomy is a preferred option which simplifies the acute management. Splenic preservation should be considered in those residing in endemic areas when facilities allow.
